# A single-arm pilot study of guided self-help treatment based cognitive behavioral therapy for bulimia nervosa in Japanese clinical settings

**DOI:** 10.1186/s13104-018-3373-y

**Published:** 2018-04-25

**Authors:** R. Setsu, K. Asano, N. Numata, M. Tanaka, H. Ibuki, T. Yamamoto, R. Uragami, J. Matsumoto, Y. Hirano, M. Iyo, E. Shimizu, M. Nakazato

**Affiliations:** 1 Department of Cognitive Behavioral Physiology, Chiba University Graduate School of Medicine, Chiba, Japan; 2 Research Center for Child Mental Development, Chiba University, Chiba, Japan; 3 Center for Forensic Mental Health, Chiba University, Chiba, Japan; 4 Institute for Psychological Research, Meiji Gakuin University, Tokyo, Japan; 5 Department of Clinical Cell Biology and Medicine, Chiba University Graduate School of Medicine, Chiba, Japan; 6 Department of Psychiatry, Chiba University Graduate School of Medicine, 1-8-1 Inohana, Chuo-ku, Chiba, Chiba 260-8670 Japan; 7 Department of Psychiatry, School of Medicine, International University of Health and Welfare, 4-3 Kozunomori, Narita City, Chiba 286-8686 Japan

**Keywords:** Eating disorders, Bulimia nervosa, Guided self-help treatments based on cognitive behavioral therapy (CBT-GSH)

## Abstract

**Objective:**

Guided self-help treatments based on cognitive behavioral therapy (CBT-GSH) are regarded as a first-line effective treatment for bulimia nervosa (BN). With limited application for CBT-GSH in Japanese clinical settings, we conducted a single arm pilot study in order to confirm the acceptability and availability of CBT-GSH in Japan.

**Results:**

25 women with BN received 16–20 sessions of face-to-face CBT-GSH. Primary outcomes were the completion rate of intervention and abstinence rates from objective bingeing and purging as assessed by the Eating Disorder Examination. Secondary outcomes were other self-report measurements of the frequency of bingeing and purging, and characteristic psychopathologies of eating disorders. Assessments were conducted before CBT as baseline as well as after CBT. 92% (23/25) of the participants completed the CBT sessions. After CBT-GSH, 40% (10/25) of the participants (intention-to-treat) achieved symptom abstinence. The mean binge and purge episodes during the previous 28 days improved from 21.88 to 10.96 (50% reduction) and from 22.44 to 10.88 (52% reduction), each (before CBT-GSH to after CBT-GSH), and the within-group effect sizes were medium (Cohen’s d = 0.67, 0.65, each). Our study provided a preliminary evidence about the feasibility of CBT-GSH in Japanese clinical settings for the future.

*Trial registration* This study was registered retrospectively in the national UMIN Clinical Trials Registry on July 10, 2013 (registration ID: UMIN000011120)

## Introduction

Bulimia nervosa (BN) is a serious psychiatric illness characterized by repetitive overeating and purging behaviors to prevent weight gain, and is commonly seen in young women. The prevalence rate of BN in Japan is estimated to be between 1.9 and 2.9% [1], and it is reported that the prevalence of BN among Japanese females has been increasing over time [2].

The treatment of BN includes psychotherapy as well as medication based mainly on antidepressants [3]. Especially, cognitive behavioral therapy (CBT), which is focused on BN, has demonstrated high efficacy [4, 5], and self-help approaches that rely on highly structured CBT treatment manuals are considered promising [5]. In fact, previous reviews have shown that CBT-based guided self-help (CBT-GSH) can be effective in treating patients with BN [6–9]. However, in Japan the penetration of both highly structured CBT and CBT-GSH is lagging behind, so patients with eating disorders (EDs) are facing the difficulty of gaining access to the best suited treatments [10].

“Getting Better Bite by Bite (GBBB)” [11] is one of the well confirmed bibliographies for a self-help program that has been evaluated by controlled trial. In previous studies, it was revealed that 7.5–30% of the patients using this manual achieved full remission from BN in both adults [12–15] and adolescents [16] at the end of treatment.

The purpose of this study was to investigate our previous study [17] with a larger number of participants and to report the acceptability and preliminary outcomes of CBT-GSH with the Japanese versions of GBBB for patients with BN in Japanese clinical settings.

## Main text

### Materials and methods

#### Study design

This study was a prospective clinical pilot study of a single-group open-label trial. The Research Ethics Committee of Chiba University Graduate School of Medicine approved all procedures. This study was registered in the national UMIN Clinical Trials Registry (ID: UMIN000011120). Written informed consent was obtained from all participants. All procedures were performed in accordance with the Helsinki Declaration. We estimated that a sample size of 18 would allow us to detect significant differences in the frequency of bingeing or purging before and after CBT-GSH with 80% power (α = 0.05) based on the mean and standard deviation of the frequency of bingeing or purging reported in previous studies with similar intervention on GBBB [14, 15]. Since 25–29% dropout rates were suggested in their studies [14, 15], we determined our sample size to be 25.

#### Participants

Inclusion criteria into this study were a primary diagnosis of BN according to DSM-IV, and age 18–39 years. Exclusion criteria were psychosis, mental retardation, pervasive developmental disorders, current high risk of suicide, substance abuse or dependence, personality disorder and unstable medical condition. We recruited 32 patients with bulimia nervosa by clinical referrals from Chiba University Hospital, as well as other local psychiatric hospitals and clinics between October 2012 and November 2015. As 6 participants met the exclusion criteria and one participant declined treatment, 25 patients were eligible for participation in the intervention. Patients were assessed by senior psychiatrists at Chiba University Hospital using the Structured Clinical Interview for DSM-IV Axis I Disorders, Research Version, Patient Edition (SCID-I/P), and all patients met the current DSM-IV criteria for BN.

#### Measurements

After enrollment, we carried out the following measures in pre-intervention and post-intervention. Current symptoms were assessed by the Eating Disorder Examination Edition 16.0D (EDE 16D) [18], the Eating Disorders Examination Questionnaire (EDE-Q) [19], the Bulimic Investigatory Test, Edinburgh (BITE) [20], the Patient Health Questionnaire (PHQ-9) [21], and the Generalized Anxiety Disorder scale (GAD-7) [22]. In addition, their height and weight were measured to calculate their body-mass index (BMI) before each CBT session.

The primary outcomes were the completion rate of intervention and abstinence rates from objective bingeing and purging as assessed by EDE 16D, a structured clinical interview. Drop-outs were defined as participants who discontinued therapy despite recommendations of the therapists and those who never returned phone calls at the end of the intervention. Remission was confirmed as no longer meeting the criteria for BN diagnosis using SCID-I/P interviews conducted by senior psychiatrists at post-intervention. EDE-Q is a well-known 36-item self-report questionnaire that assesses the frequency of abnormal eating behaviors and eating disorder-related symptom profile. EDE-Q includes four subscale scores—restricting (EDE-Qr), eating concern (EDE-Qe), shape concern (EDE-Qs), and weight concern (EDE-Qw). Bingeing BITE is a 33-item self-report questionnaire designed to assess symptoms of bulimia or binge eating. BITE is composed of two subscales: the symptom scale (BITE-sas) and the severity scale (BITE-ss). The former measures the degree of symptoms present, and the latter provides an index of the severity of bingeing and purging behaviors as defined by their frequency. PHQ-9 is an easy-to-use screening tool for assessing current depressive symptoms and GAD-7 is a simple assessment tool to measure a participant’s current anxiety.

#### Interventions

Our CBT-GSH intervention was conducted in 16 weekly 50-min face-to-face sessions, which could be extended to as many as 20 sessions depending on the therapeutic need or participant’s request. Termination of intervention within at least 8 sessions was also permitted in case of symptom abstinence.

As for bibliotherapy, the self-help manual “Getting better bite by bite” [11] was used. The contents of the sessions were tailored for each participant according to their specific difficulties. Structured diagnostic interviews were conducted and self-report questionnaire were completed following the completion of the CBT intervention.

CBT-GSH was conducted by 7 therapists (3 clinical psychologists, 2 psychiatrists, 1 nurse, and 1 psychiatric social worker) who were experienced in the use of CBT-GSH for EDs and had completed the 2 year CBT training program at Chiba University [17]. All therapists attended the workshop for CBT-GSH for BN presented by Prof. Ulrike Schmidt at Chiba University in 2013. All therapists attended weekly group supervision sessions with other therapists as well as supervision sessions with a senior supervisor. Concomitant treatment with medications of equable dosage was permitted throughout the study.

#### Statistical analyses

Data analyses were carried out with SPSS version 21 (IBM Corp., Armonk, NY, USA). We used within-group t-tests to compare pre- and post-CBT-GSH. Effect size was reported by Cohen’s d. Statistical significance was designated as P < 0.01. For the participants who dropped out, the data gathered at their final visits was used for analysis.

### Results

#### Characteristics of participants and completion rate

32 patients with BN were enrolled, 26 proved eligible for the study, and 25 agreed to embark upon the treatment. Two participants discontinued therapy while remaining symptomatic (4th session and 5th session, respectively), and thus 23 participants (92%) completed the CBT sessions.

The demographic and clinical characteristics of 25 participants are summarized in Table 1. The participants were all female native Japanese speakers, between age 18 and 36 (mean = 25.60, SD = 5.52 years). All participants met the principal DSM-IV diagnostic criteria for BN. 21 (84%) of the patients practiced self-induced vomiting; eight (32%) also took laxatives to influence their shape and weight. Four patients (16%) neither vomited nor took laxatives but restricted their eating or exercised sufficiently to meet the criteria (purging type, 21; non-purging type, 4). Additional Axis I diagnoses for the patients included major depressive disorder (36%), other mood disorders (e.g., dysthymic disorder, 4%; bipolar II disorder, 12%) and other anxiety disorders (e.g., obsessive–compulsive Disorder, 4%; panic disorder, 4%). Seven patients had been on medication, and 10 patients had a history of anorexia nervosa. Other demographic and clinical variables of the participants are shown in Table 1.

**Table 1:** Demographic and clinical data of the patients with bulimia nervosa

	Bulimia nervosa		Range
	(n = 25)		
	Mean	SD	
Age (years)	25.60	5.52	(18.2–36.9)
Duration of ED (years)	5.00	4.49	(0.3–15.4)
Education (years)	14.14	2.16	(9–18)
BMI (kg/m^2^)	20.34	2.34	(17.9–24.6)
Gender (female, n, %)	25 (100.0)		
Comorbidity (n, %)			
Major depressive disorder	9 (36.0)		
Dysthymic disorder	1 (4.0)		
Bipolar II disorder	3 (12.0)		
Obsessive–compulsive disorder	1 (4.0)		
Panic disorder	1 (4.0)		
Medication (n, %)			
Medication-free	18 (72.0)		
SSRI monotherapy	4 (24.0)		
SSRI and topiramate	1 (4.0)		
SSRI and ethyl loflazepate	1 (4.0)		
Topiramate and ethyl loflazepate	1 (4.0)		
Past history (n, %)			
Anorexia nervosa	10 (40.0)		
Major depressive disorder	3 (12.0)		

*ED* eating disorders, *SD* standard deviation, *BMI* body mass index, *SSRI* selective serotonin reuptake inhibitors

#### Treatment outcomes

The average number of sessions per participant (completer) was 18.83 (SD = 3.38). Table 2 shows the abstinence rates for patients with BN on EDE 16D pre- and post-CBT-GSH. 10 participants (40%) achieved all-symptom abstinence and 13 participants (52%) met the criteria for remission by DSM-IV at the end of treatment.

**Table 2:** Abstinence rates from objective bingeing, purging assessed by EDE 16D between pre and post CBT-GSH

	Pre CBT-GSH (n = 25)		Post CBT-GSH (n = 25)	
	N	%	N	%
Objective bingeing				
Abstinence	0	0.0	10	40.0
Subclinical	1	4.0	4	16.0
Clinical	24	96.0	9	36.0
Drop-outs	–	–	2	8.0
Vomiting				
Abstinence	4	16.0	12	48.0
Subclinical	1	4.0	4	16.0
Clinical	20	80.0	7	28.0
Drop-outs	–	–	2	8.0
Bingeing or purging (at least one symptom)				
Abstinence	0	0.0	10	40.0
Subclinical	0	0.0	3	12.0
Clinical	25	100.0	10	40.0
Drop-outs	–	–	2	8.0

*CBT*-*GSH* guided self-help treatments based on cognitive behavioral therapy, *EDE 16D* Eating Disorder Examination Edition 16.0D, “abstinence”, behavior absent during the previous 28 days; “subclinical”, behavior present during previous 28 days less than twice per week; “clinical”, behavior present during previous 28 days two or more times per week

Table 3 shows the changes in the number of binge and purge episodes during the previous 28 days as well as other clinical self-report measures during CBT-GSH. The mean binge episodes improved from 21.88 to 10.96 (50% reduction, before CBT-GSH to after CBT-GSH), and the within-group effect size at the end-point assessment was medium (Cohen’s d = 0.67). The mean purge episodes improved from 22.44 to 10.88 (52% reduction), and the within-group effect size was medium (Cohen’s d = 0.65). Other outcomes are also shown in Table 3. The CBT-GSH intervention led to significant reductions in all outcome measures at the end of treatment (pre–post CBT; P < 0.001).

**Table 3:** Changes in the frequencies of bingeing and purging, psychological tests between pre and post CBT-GSH (n = 25)

	Pre CBT-GSH		Post CBT-GSH^c^		*P*	Effect size	
	Mean	SD	Mean	SD		d	(95% CI)
Objective binge days^a^ (EDE 16D)	17.56	9.93	8.48	10.73	< 0.001*	0.88	(0.30, 1.46)
Purge days^a^ (EDE 16D)	17.24	11.31	9.44	11.81	0.001*	0.67	(0.10, 1.25)
Frequency of binge episodes^b^ (EDE-Q item 17)	21.88	15.69	10.96	17.12	0.001*	0.67	(0.09, 1.24)
Frequency of purge episodes^b^ (EDE-Q item 22)	22.44	19.32	10.88	16.35	0.001*	0.65	(0.08, 1.22)
BMI	20.34	2.34	20.63	2.10	0.280	− 0.13	(− 0.68, 0.42)
EDE-Qg	4.10	1.27	2.49	1.57	< 0.001*	1.13	(0.53, 1.73)
EDE-Qr	3.54	1.70	1.86	1.85	< 0.001*	0.95	(0.36, 1.54)
EDE-Qe	3.93	1.42	2.03	1.69	< 0.001*	1.22	(0.61, 1.83)
EDE-Qw	4.38	1.27	2.91	1.59	< 0.001*	1.02	(0.43, 1.61)
EDE-Qs	4.54	1.28	3.16	1.64	< 0.001*	0.94	(0.35, 1.53)
BITEss	11.84	3.09	5.40	4.72	< 0.001*	1.61	(0.97, 2.25)
BITEsas	23.84	4.95	14.48	8.40	< 0.001*	1.36	(0.74, 1.98)
PHQ-9	13.92	6.75	6.28	4.90	< 0.001*	1.30	(0.68, 1.91)
GAD-7	8.68	5.19	4.16	3.79	< 0.001*	0.99	(0.41, 1.58)

*CBT*-*GSH* guided self-help treatments based on cognitive behavioral therapy, *SD* standard deviations, *d* Cohen’s d, *EDE 16D* Eating Disorder Examination Edition 16.0D, *EDE*-*Q* Eating Disorder Examination Questionnaire, *BMI* body mass index, *BITE* Bulimic Investigatory Test; Edinburgh, *PHQ*-*9*, 9-item patient health questionnaire, *GAD*-*7* 7-item generalized anxiety disorder scale

* P < 0.05, within-group t-tests

^a^Number of symptomatic days during the previous 28 days

^b^Number of symptomatic episodes during the previous 28 days

^c^The presented date includes the data gathered from 2 dropped out participants at the final visit

### Discussion

This study confirmed the acceptability of face-to-face CBT-GSH. 96% of the eligible participants agreed to embark upon the treatment, and 92% carried on to its completion. In previous studies using GBBB, it was suggested that retention rates at the end of treatment were in the range of 71.0–86.3% [12–15]. Our result is comparable to previous clinical trials of CBT-GSH [12–15]. Secondly, 40% of the participants (intention-to-treat) achieved symptom abstinence, and the number of binge and purge episodes improved significantly together with a decline in other eating and general psychopathology. Since previous studies using GBBB revealed that 7.5–30% of the patients achieved full remission from BN [12–16], our result is at least comparable to those studies.

The recent questionnaire survey of children and youth in schools revealed high rates of suspected cases of BN and binge eating disorders (BED) among adolescents, but there were few chances to access special treatment services due to the hesitation of the patients as well as the lack of available delivery services in EDs in the community [23]. A way of providing support in the community should be required, as the proportion of teens at the estimated onset age of EDs has increased year by year in Japan [24]. Improving delivery of best evidence-based intervention for patients with EDs is one of the priority issues to be solved in Japan [2]. One of the advantages of self-help interventions is that they can be implemented by a wide range of health care providers [25]. Since the therapists from various professional backgrounds demonstrated the high acceptability and good therapeutic outcomes of CBT-GSH, our study indicated favorable accessibility of the CBT-GSH in Japanese clinical settings.

### Conclusions

Our study provided a preliminary evidence about the feasibility of CBT-GSH in Japanese clinical settings for the future.

## Limitations

The limitations arising from the design of the present study include the small sample size, a single-arm design with no control group, a single-site study, and a lack of follow-up data. Further study will be required using a randomized controlled trial across longer follow-up.

## Abbreviations

CBT-GSH: guided self-help treatments based on cognitive behavioral therapy
BN: bulimia nervosa
EDs: eating disorders
CBT: cognitive behavioral therapy
DSM-IV: Diagnostic and Statistical Manual of Mental Disorders, 4th Edition
SCID-I/P: Structured Clinical Interview for DSM-IV Axis I Disorders, Research Version, Patient Edition
GBBB: “Getting Better Bite by Bite”
EDE 16D: Eating Disorder Examination Edition 16.0D
EDE-Q: Eating Disorders Examination Questionnaire
EDE-Qg: Eating Disorders Examination Questionnaire global score
EDE-Qr: Eating Disorders Examination Questionnaire restricting score
EDE-Qe: Eating Disorders Examination Questionnaire eating concern score
EDE-Qs: Eating Disorders Examination Questionnaire shape concern score
EDE-Qw: Eating Disorders Examination Questionnaire weight concern score
BITE: Bulimic Investigatory Test, Edinburgh
BITE-sas: Bulimic Investigatory Test, Edinburgh, the symptom scale
BITE-ss: Bulimic Investigatory Test, Edinburgh, severity scale
PHQ-9: 9-item patient health questionnaire
GAD-7: 7-item generalized anxiety disorder scale
BMI: body mass index

## References

[1] Chisuwa N, O’Dea JA (2010). Body image and eating disorders amongst Japanese adolescents. A review of the literature. Appetite..

[2] Nakai Y, Nin K, Noma S (2014). Eating disorder symptoms among Japanese female students in 1982, 1992 and 2002. Psychiatry Res.

[3] NICE. Eating disorders: recognition and treatment|Guidance and guidelines|NICE. 2017. https://www.nice.org.uk/guidance/ng69/chapter/Recommendations#treating-bulimia-nervosa. Accessed 22 Feb 2018.

[4] Bacaltchuk J, Hay P, Trefiglio R (2001). Antidepressants versus psychological treatments and their combination for bulimia nervosa. Cochrane Database Syst Rev..

[5] Hay PP, Bacaltchuk J, Stefano S, Kashyap P (2009). Psychological treatments for bulimia nervosa and binging. Cochrane Database Syst Rev..

[6] Perkins SJ, Murphy R, Schmidt U, Williams C (2006). Self-help and guided self-help for eating disorders. Cochrane Database Syst Rev..

[7] Sysko R, Walsh BT (2008). A critical evaluation of the efficacy of self-help interventions for the treatment of bulimia nervosa and binge-eating disorder. Int J Eat Disord.

[8] Sanchez-Ortiz VC, Schmidt U, Grilo CM, Mitchell JE (2010). Self-help approaches for bulimia nervosa and binge-eating disorder. The treatment of eating disorders.

[9] Wilson GT, Zandberg LJ (2012). Cognitive-behavioral guided self-help for eating disorders: effectiveness and scalability. Clin Psychol Rev..

[10] Nishizono-Maher A (2013). Eating disorder treatment now; the NICE guidelines for practice in Japan.

[11] Schmidt U, Treasure J, Alexander J (1993). Getting better bit(e) by bit(e): a survival kit for sufferers of bulimia nervosa and binge eating disorders.

[12] Treasure J, Schmidt U, Troop N, Tiller J, Todd G, Keilen M (1994). First step in managing bulimia nervosa: controlled trial of therapeutic manual. BMJ.

[13] Treasure J, Schmidt U, Troop N, Tiller J, Todd G, Turnbull S (1996). Sequential treatment for bulimia nervosa incorporating a self-care manual. Br J Psychiatry J Ment Sci..

[14] Thiels C, Schmidt U, Treasure J, Garthe R, Troop N (1998). Guided self-change for bulimia nervosa incorporating use of a self-care manual. Am J Psychiatry.

[15] Bailer U, de Zwaan M, Leisch F, Strnad A, Lennkh-Wolfsberg C, El-Giamal N (2004). Guided self-help versus cognitive-behavioral group therapy in the treatment of bulimia nervosa. Int J Eat Disord.

[16] Schmidt U, Lee S, Beecham J, Perkins S, Treasure J, Yi I (2007). A randomized controlled trial of family therapy and cognitive behavior therapy guided self-care for adolescents with bulimia nervosa and related disorders. Am J Psychiatry.

[17] Kobori O, Nakazato M, Yoshinaga N, Shiraishi T, Takaoka K, Nakagawa A (2014). Transporting Cognitive Behavioral Therapy (CBT) and the Improving Access to Psychological Therapies (IAPT) project to Japan: preliminary observations and service evaluation in Chiba. J Ment Health Train Educ Pract..

[18] Fairburn CG, Cooper Z, O’Connor M, Fairburn CG (2008). Eating disorder examination (16.0D). Cognitive behavior therapy and eating disorders.

[19] Fairburn CG, Beglin SJ (1994). Assessment of eating disorders: interview or self-report questionnaire?. Int J Eat Disord.

[20] Henderson M, Freeman CP (1987). A self-rating scale for bulimia. The “BITE”. Br J Psychiatry J Ment Sci..

[21] Kroenke K, Spitzer RL, Williams JB (2001). The PHQ-9: validity of a brief depression severity measure. J Gen Intern Med.

[22] Spitzer RL, Kroenke K, Williams JBW, Löwe B (2006). A brief measure for assessing generalized anxiety disorder: the GAD-7. Arch Intern Med.

[23] Seike K, Nakazato M, Hanazawa H, Ohtani T, Niitsu T, Ishikawa S-I (2016). A questionnaire survey regarding the support needed by Yogo teachers to take care of students suspected of having eating disorders (second report). Biopsychosoc Med..

[24] Ministry of Health, Labor and Welfare—Japan. Eating disorders. https://www.e-healthnet.mhlw.go.jp/information/heart/k-04-005.html (**in Japanese**).

[25] Latner J, Wilson GT (2007). Self-help for obesity and eating disorders.

